# Care burden, coping styles and involvement in care in mothers of autistic children in pandemic of COVID‐19

**DOI:** 10.1002/nop2.1256

**Published:** 2022-06-17

**Authors:** Fateme Mohammad, Niloofar Sani, Khodayar Oshvandi, Seyedeh Zahra Masoumi, Salman Khazaei, Saeid Bashirian, Ensiyeh Jenabi, Majid Barati, Mohammad Rezaei, Seyed Reza Borzou

**Affiliations:** ^1^ Chronic Diseases (Home Care) Research Center and Autism Spectrum Disorders Research Center Department of Nursing Hamadan University of Medical Sciences Hamadan Iran; ^2^ Student Research Center Hamadan University of Medical Sciences Hamadan Iran; ^3^ Department of Medical Surgical Nursing School of Nursing and Midwifery Mother and Child Care Research Center Hamadan University of Medical Sciences Hamadan Iran; ^4^ Department of Midwifery School of Nursing and Midwifery Mother and Child Care Research Center Hamadan University of Medical Sciences Hamadan Iran; ^5^ Department of Public Health Department of Epidemiology School of Health, Social Determinants of Health Research Center Health Sciences & Technology Research Institute Hamadan University of Medical Sciences Hamadan Iran; ^6^ Department of Public Health School of Health Social Determinants of Health Research CenterHealth Sciences & Technology Research Institute Hamadan University of Medical Science Hamadan Iran; ^7^ Autism Spectrum Disorders Research Center Hamadan University of Medical Sciences Hamadan Iran; ^8^ Department of Public Health School of Health, Autism Spectrum Disorders Research Center Hamadan University of Medical Sciences Hamadan Iran; ^9^ Department of Speech Therapy School of Rehabilitation Sciences, Autism Spectrum Disorders Research Center Hamadan University of Medical Sciences Hamadan Iran; ^10^ Department of Medical Surgical Nursing School of Nursing and Midwifery, Chronic Diseases (Homecare) Research Center Hamadan University of Medical Sciences Hamadan Iran

**Keywords:** autistic children COVID‐19, care burden, coping styles, involvement in care, mothers

## Abstract

**Aim:**

The aim of the present study was to investigate the burden of care, coping styles and involvement in the care of mothers of autistic children in the pandemic of COVID‐19 in Iranian society.

**Design:**

A cross‐sectional study.

**Methods:**

A total of 134 mothers completed questionnaires online. Data were analysed by descriptive statistics (frequency, percentage, mean and standard deviation) and independent *t*‐test, ANOVA and multiple linear regressions. The significance level was considered *p* < .05.

**Result:**

Findings of the study found that burden of care has a strong and direct correlation with involvement in care (*p* < .001, *r* = .78) and strongly and indirectly correlated with coping styles (*p* < .001, *r* = −.82). Variables of coping styles, involvement in care, mothers' occupation and number of children, age and functional level of autism can predict 81.27% of the variance in care burden in these mothers.

## 1 BACKGROUND

Autism is one of the most important developmental–behavioural disorders in the last decade that has growth more than 6% all of the world, and has been caused a large number of families all of the world confronting to the challenges of caring for these children (Feldman & Werner, 2002; Sarabi, 2011). This issue will face organizations providing healthcare services to many challenges in the near future (McGuire, 2016). Because children with autism have many problems in cognition, movement and interaction (Greenspan & Wieder, 2006) that affect the behavioural development, verbal development and social interactions of these children in different situations (Ingersoll & Hambrick, 2011; Suzuki et al., 2015).

Inability for self‐care and dependence on the caregivers are the most important problems in children with autism (Bal et al., 2015; Jasmin et al., 2009). This subject causes caring of children with autism require a lot of time and energy and subsequently imposes a lot of physical–psychological steers and height caring burden to caregivers in the long time (Al‐Farsi et al., 2016; Jenaro et al., 2020).It is clear that parents, and especially mothers, are the important caregivers of children with autism who are in a difficult situation due to many problems of these children and bear a heavy care burden that affects various aspects of their life (Greenspan & Wieder, 2006).

The care burden is referred to a set of psychological, emotional, social and economic challenges experienced by the caregiver of physical and mental diseases that leads to psychological problems, poor quality of life, low energy level, fatigue and physical disorders (Akram et al., 2019). However, caring for children with autism is difficult and tedious and requires a structured program and a lot of energy and time (Mohammadi et al., 2020). Therefore, caring for and educating these children imposes a lot of care burden on mothers (Mohammadi et al., 2018). The care burden imposed on the mothers of children with autism has led them to find strategies to cope with existing conditions in order to control caring stresses and cope with existing conditions (Brown et al., 2020; Dobre & Topală, 2020). There are two general types of problem‐based coping in these mothers (strategies aimed at solving a problem or doing something to change stress) and emotion‐based coping (strategies aimed at reducing or managing the anxiety caused by stressors) (Bozkurt et al., 2019). These strategies have been identified as important mechanisms in controlling stress, fatigue, care burden and improving health level of these mothers (Furrukh & Anjum, 2020), which causes to promote the mothers' involvement in the care of these children. Parental involvement is one of the key elements in providing quality care (Ygge & Arnetz, 2004), ranging from the passive presence of parents to full involvement in care (Abdelkader et al., 2016). However, parental involvement in child care is considered as one of the major challenges for healthcare providers, who seek to enhance parental competence, self‐confidence and improve mother–child interactions (Aein et al., 2009). About this matter that children with autism need the basic cares of parents, especially mothers, it is important to examine the factors affecting parental involvement in caring of these children (Wang et al., 2020). However, fathers are often less involved in caring for their children due to work commitments (Rankin et al., 2019), so research shows that fathers of children with autism spend less time to care their children (Wang et al., 2020). While the studies have shown that there is a statistically significant negative relation between fathers' involvement and mothers stress in families having children with autism. (DeMontigny et al., 2020; Hu et al., 2017). However, the emerging disease of COVID‐19 can affect the care burden and coping styles methods of these parents.

The experience of being quarantine during of COVID‐19 has caused a wide range of psychological problems in a statistically significant number of people. It seems that having a psychiatric background during quarantine leads to worse and more unpleasant results, and these people need more support during quarantine (Alhuzimi, 2021). Meanwhile, it seems that COVID‐19 pandemic severely affect people with special needs, including children with autism and their families, so that parents of children with autism during the COVID‐19 crisis are probably to bear high‐care burden due to ambiguous economic situation, limited access to treatment and medical procedures and long delays in accessing care programs and experience more stress and decrease their capacity to care for their children (Lin et al., 2020).

However, one of the most important duties of paediatric nurses is support and educates the child and their family. Meanwhile, children with autism and their families are one of the most important groups that needing to paediatric nurses services. Parents of these children usually turn to paediatric nurses for behavioural counselling, coping with the disease of autistic child, controlling stress and parenting tensions, educating these children, reduce care burden and creating stability in the family. So that these mothers can better understand the behavioural and developmental conditions and characteristics of their autistic children and control and manage their parenting stress (Alhuzimi, 2021; Furrukh & Anjum, 2020).

Mothers of children with autism experienced countless challenges in caring of children with autism and have reported a high burden of care that severely was affected their ability and tolerance. On the other hand, due to the lack of immediate effect of education and appropriate and continuous changes in the behaviour of autistic children, these mothers become frustrated and abandon the education and care of the child and do not participate in the continuous education of their children. Withdrawing from active participation in the education of an autistic child causes they experience inefficiency and lack of parental competence much more and cannot adapt to the child's disease. This is while the closure of autism centres in Iran has imposed a greater burden of care on mothers, which can affect their adaptation and participation in the care of these children (Mohammadi et al., 2020).

Therefore, due to the close and reciprocal relationship of the care burden with coping strategies and involvement in caring mothers of children with autism, we decided to design and implement this study. The purpose of this study was to “investigate the relationship between care burden with coping styles, involvement in the care and demographic characteristics in in mothers of autistic children.” Also, the research hypothesis was there is relationship between care burden with coping styles, involvement in the care and demographic characteristics in in mothers of autistic children”.

## 2 METHODS

### 2.1 Study design and setting

This study employed a cross‐sectional research design. The conducted investigation is based on the strengthening the reporting of observational studies in epidemiology statement (STROBE), that is checklist for observational research, from April 2021 to August 2021. The two following aims were examined in study “evaluation of care burden, coping styles and involvement in the care in mothers of autistic children” and “investigating the relationship between care burden, coping styles, involvement in the care and demographic characteristics in mothers of autistic children.”

### 2.2 Participants and sampling

For sampling, the correspondence author went to three autism centres affiliated with university of medical sciences in the west of Iran, after receiving ethical approval and got the phone number of mothers of children with autism that had the inclusion criteria. Then, the correspondence called the mothers, if they tended to participate in this study, took their emails and emailed the questionnaires to them. Therefore, 134 of mother with autistic children were invited via email and selected through convenience sampling to participate in the study.

Inclusion criteria were as follows: age between 6 and 16 years old; high or moderate performance based on the psychiatrist's report and the diagnostic and statistical manual of mental disorders (DSM V) guideline; no other physical, cognitive‐developmental or mental disorder and the desire and consent of their parents to participate in this study. The participants who failed to answer more than half of the items on their questionnaires or did not return their questionnaires were excluded. The researchers sent emails and reminder messages to the participants; so that the majority of the questionnaires (90%) were completely gathered in August. Faintly, 110 mothers completed and returned the questionnaires via e‐mail. Thus, the response rate was 82.089%, the mothers' reasons for not being participated in this study were not having time to fill out questionnaires and being busy.

### 2.3 Questionnaire

#### 2.3.1 Demographic information questionnaire

Demographic information included age, sex, economic status, number of children, number of sick children, number of children with autism and their severity of autism, parents' age, parents' educational level, parents' occupation and living with parents.

#### 2.3.2 Burden assessment scale (BAS)

The burden assessment scale (1994) has been designed by Reinhard and Horowitz. This questionnaire assesses care burden in two dimensions physical and mental in caregivers of people with neuropsychiatric disorders and consists19 items which is graded according to the four‐point Likert scale from high burden (score 4) to any burden (score 1). Higher scores indicate more care burden. Face, content validity and reliability of this scale were examined in the study of Reinhard et al. (1994), so that the face and content validity was appropriate and the reliability of this scale was estimated 0.89% by Cronbach's alpha method (Reinhard et al., 1994).

#### 2.3.3 Parental bonding instrument (PBI)

The parental bonding instrument was developed in 1979 by Parker et al and is used to assess parental involvement in caring for their child. This scale includes 25 items in two dimensions, caring (12 questions) and extreme support (13 questions). It is scored on a 4‐point Likert scale (very high: 0 to very low: 3). It should be noted that 1, 5, 6, 8–13, 17, 19 and 20 items are scored conversely. Higher scores indicate a positive bonding of the child with the parent. Face and content validity and the reliability of this scale were examined in the study of Mohebbi (2019), so that it has a very good internal homogeneity with a reliability coefficient of 0.88 for the care subscale and 0.74 for the extreme support subscale by split‐half method (Mohebbi, 2019).

#### 2.3.4 Coping strategies questionnaire (CSQ)

The coping strategies questionnaire (1980) was designed by Lazarus & Folkman. It is a tool to study how people cope with tensions. This questionnaire consists of 66 questions and has 8 dimensions (direct coping, avoidance, self‐control, seeking social support, responsibility, escape and avoidance, managerial problem‐solving and positive re‐evaluation). Questions are scored on a 4‐point Likert scale (from score 0: I have not used at all to score 3: I use a lot). In this questionnaire, if the calculated score is between 0 and 66, it is a sign of using low level of coping style in the person, if the calculated score is between 66 and 110, it is a sign of using moderate level of coping style in the person, and finally, if the calculated score is 110 or higher, it is a sign of using high level of coping style in the person. Face and content validity and reliability of this scale were examined in Attaran study in 2012, so that face and content validity is appropriate and the reliability of this questionnaire has been in scales from 0.61 to 0.79 by Cronbach's alpha method (Attaran, 2013).

### 2.4 Statistical methods

In this study, the collected data were analysed with SPSS software version 22. For this purpose, descriptive statistics (frequency, percentage, mean and standard deviation) were used. Independent *t*‐test and ANOVA were also used to investigate the relationship between caring burden and demographic information. The significance level was considered *p* < .05. Then demographic information, coping strategies and parental bonding (*p* < .25) were entered into the multiple linear regression model with a backward strategy. In addition, comparing the results of simple and multiple linear regressions we investigated the effect of moderator variables on the relationships between caring burden with demographic information, coping strategies and parental bonding. The researcher evaluated before performing multiple linear regression, hypotheses including normality of data, homogeneity of variance and independence of the residual.

### 2.5 Ethics approval and consent to participate

The study design was approved by the ethics committee of the Hamadan University of Medical Sciences (1,400.171). Also at the beginning of study the researcher introduced herself and explained the goals of the study and assured that all information would remain confidential and that they could withdraw from the study at any time. Finally, the written informed consent was obtained from all the participants after providing them with sufficient information on the study.

## 3 RESULTS

### 3.1 Demographic information

Of the 110 mothers who participated in the study. The range of the participants' ages was between 24 and 55 years with the mean of 34.74 ± 3.16 years. The majority of the participants 88.18% were married, 40.90% of whom had two children. Also, most of the participants had a diploma 59.10%, were housewife 50. % and had boy child 70.90%.

### 3.2 The relationship between caring burden and demographic information

The findings of the study found that there was a statistically significant relationship between caring burden with mother's jab, number of children, children's age and level of autism in children. So that, employee mothers with 3 or more children that had children with low‐functioning autism and autistic children age 12–14 years reported more care burden (Table 1).

**TABLE 1:** Participants' demographic characteristics and caring burden scores

Demographic variables	Number (%)	Occupational burnout Means ±SD	*p*. value
Mother's age (Year)			
24–33	31 (19.10)	65 ± 1.31	.721^**^
34–44	72 (65.45)	64 ± 1.63	
45–55	17 (15.45)	64 ± 1.57	
Mother's education			
Illiterate	3 (2.73)	62 ± 1.26	.892^**^
Primary	7 (6.37)	62 ± 1.78	
Diploma	65 (59.10)	62 ± 1.43	
Bachelor	23 (20.90)	62 ± 2.01	
Master's degree and higher	12 (10.90)	62 ± 2.42	
Mother's Jab			
Self‐employed	31 (28.18)	61 ± 1.65	.021^**^
Employee	24 (21.82)	69 ± 1.39	
Housewife	55 (50.00)	60 ± 1.37	
Marital status			
Married	97 (88.18)	62 ± 2.01	.82^*^
Divorce	13 (11.82)	63 ± 1.72	
Number of children			
1	38 (34.55)	64 ± 1.97	.017^**^
2	45 (40.90)	69 ± 1.31	
3 and more	27 (24.55)	73 ± 1.32	
Number of children with autism			
1	96 (87.27)	69 ± 1.47	.92^*^
2	14 (12.73)	70 ± 1.87	
Sex of children			
Boy	78 (70.90)	67 ± 1.53	.77^*^
Girl	32 (29.10)	69 ± 1.97	
Children's age			
6–8	37 (33.64)	67 ± 1.67	.018^**^
9–11	47 (42.73)	71 ± 1.24	
12–14	26 (23.63)	74 ± 1.86	
Level of autism in children			
High performance	58 (61.82)	64 ± 1.76	.012^*^
Low performance	42 (38.18)	72 ± 1.38	

^*^ Independent *t*‐test.

^**^ ANOVA test.

### 3.3 Caring burden, coping strategies and parental bonding in the participants

The caring burden means score of the mothers who participated in the present study was 64.74 ± 2.57, the coping strategies means score was 68.81 ± 2.76. Also, the parental bonding means score was found to be 45.51 ± 1.88 during the COVID‐19 crisis (Table 2).

**TABLE 2:** Means and standard deviations of the participants' caring burden, coping strategies and parental bonding scores

Variable	Dimension	Means ± SD Per dimension	Total Means ± SD
Caring burden	Physical	64.32 ± 1.88	64.74 ± 2.57
	Mental	65.16 ± 2.63	
Coping strategies	Direct confrontation	67.31 ± 3.22	68.81 ± 2.76
	Self‐control	69.98 ± 2.13	
	Seeking social	68.87 ± 3.41	
	Support	71.21 ± 2.33	
	Responsibility	70.24 ± 2.61	
	Avoidance	68.18 ± 2.27	
	Problem‐solving	66.31 ± 3.45	
	Positive re‐evaluation	68.42 ± 2.33	
Parental bonding	Caring	44.31 ± 1.42	45.51 ± 1.88
	Extreme support	46.71 ± 1.78	

### 3.4 The relationship between caring burden, coping strategies and parental bonding in the participants

The findings of the study show that there is a strong and direct correlation between caring burdens with parental bonding in mothers of children with autism in the pandemic of COVID‐19 (*p* < .001, *r* = .78).Also, a more strong and indirect correlation was found to exist between caring burden with coping strategies (*p* < .001, *r* = −.82).

### 3.5 The predictor variables of caring burden in mothers of children with autism in the pandemic of COVID‐19

The variable of coping strategies, parental bonding, mother's jab, number of children, children's age and level of autism in children which had a *p*‐value of smaller than .25 were entered into multiple linear regressions with the backward technique. These variables remained in the model and accounted for about 81.27% of the caring burden variance in the mothers of autistic children in the pandemic of COVID‐19 (Table 3).

**TABLE 3:** Predictor variables of caring burden in mothers of children with autism in pandemic COVID‐19

Variable	Unstandardized coefficients		Standardized coefficients	T	*p*‐value
	B	Standard deviation	*β*		
Coping strategies	−0.744	2.62	−.794	−3.42	.001
Parental bonding	0.708	2.21	.768	3.28	.001
Mother's jab	0.313	2.27	.377	2.98	.031
Number of children	0.295	2.76	.310	3.21	.034
Children's age	0.642	2.31	.661	1.87	.042
Level of autism in children	0.651	1.53	.672	1.43	.039
Adjusted R2=81.27%					

## 4 DISCUSSION

This study found mothers of children with autism have reported high levels of caring burden, moderate levels coping strategies and high levels of parental bonding. There was a strong and indirect correlation between caring burdens with coping strategies but strong and direct correlation between caring burdens with parental bonding. In addition, the results found that caring burden in mothers of children with autism correlates with their coping strategies, parental bonding and mother's job, number of children, children's age and level of autism in children. These variables predicated 81.27% of caring burden variance in these mothers.

Although a few studies have examined the work stress, knowledge and awareness of mothers with autistic children in during COVID‐19, no article has been found that investigating relationship between caring burden with coping strategies, parental bonding and demographic information in these mothers. Therefore, the researchers used other articles that measured the level of caring burden, coping strategies and parental bonding in mothers with autistic children separately before incidence COVID‐19.

The care burden score reported by mothers of children with autism as the primary caregiver of these children in this study was 2.57 ± 64.74, which indicates the high care burden that mothers bear in caring for these children. However, the care burden is one of the most challenging issues in the care and maintenance of these children. The findings of this study show that the number of children, maternal occupation, age and performance level of children with autism strongly affect the care burden imposed on these mothers. In line with the findings of this study, other studies have shown that parents, especially mothers of children with autism, have reported a high care burden for these children (Bozkurt et al., 2019; Marsack‐Topolewski et al., 2021; Marsack‐Topolewski & Maragakis, 2021). Marsack‐Topolewski et al. (2021) also stated that mothers of children with autism have reported a high‐care burden for these children. They also stated that the severity of autism disorder and subsequently, behaviours and care needs of these children significantly affect the care burden imposed on these parents, which is consistent with the present study (Marsack‐Topolewski & Maragakis, 2021). Although Bozkurt et al. (2019) also stated in their study that parents of children with autism experienced a high‐care burden that is consistent with the present study, they reported that care burden has been more on parents with one child compared to several children and having a girl compared with a boy, which is contrary to the above study (Bozkurt et al., 2019). This difference may be due to diversity in the cultural and supportive context governing the two societies. Because in Iran, due to unfavourable economic conditions, it is not possible to give wide and comprehensive support from these parents, who may affect the severity of the care burden imposed on these parents. Consistent with the findings of this study, Marsack‐Topolewski et al. (2021) reported a higher care burden for children with autism and stated that care burden for these children depends on their ability to perform daily activities and self‐care. So that, whatever developmental disorders in children are more, their dependent on parents and subsequently care burden reported by the parents of these children will be higher.

On the other hand, in this study, care burden score was reported high and coping strategies score was reported moderate, and there was a strong and inverse relationship between care burden and coping strategies in mothers of children with autism. Consistent with the findings of this study, Bozkurt et al. (2019) stated that there is a strong and inverse relationship between care burden and coping strategies in the parents of children with autism, so that, whatever the care burden was high, the coping strategy was weaker and coping strategies were explained and described 42% variance of care burden imposed on the parents of autistic children (Bozkurt et al., 2019). The reason for this similarity could be the use of the same tools to assess coping strategies in mothers of autistic children. Ang and Loh (2019) also stated in their study that parents' coping of autistic children was strongly and inversely related to the rate of behavioural problems and the severity of autism in their children. So that, whatever children's behavioural problems has been more, the parents have had more stress and depression and less coping; and stress and coping strategies have explained 51% of mental disorders and depression in these parents. This funding is Consist with the present study (Ang & Loh, 2019). On the other hand, Samadi (2020) also reported moderate coping and good‐feeling in parents of autistic children and stated that moderate coping due to behavioural problems, conditions of autistic children and poor formal and informal support from these parents in Iranian society has caused that good‐feeling has been extremely moderate in these parents, which is in line with the present study (Samadi, 2020).

However, in the present study, the coping score in parents of autistic children has been reported lower than similar studies. This difference could be due to the occurrence of the COVID‐19 pandemic and imposing the traffic restrictions and quarantine. Because all educational centres for autistic children in Iran are closed and there are work restrictions, sometimes parents even prefer to keep their children at home because of COVID‐19, but these restrictions have caused the mothers to involve with autistic children all day and night and impose higher care burden for parents, and they have less time for their other tasks and unconsciously their coping becomes less.

In the present study, the score of mothers' involvement in the care of autistic children has been reported high and there was a strong and direct relationship between care burden and mothers' involvement in the care of autistic children. Consistent with the present study, several studies have said that the involvement of mothers with autistic children in the care of these children is high (Flippin & Crais, 2011; Mello et al., 2019; Mo et al., 2020). In this regard, Wang et al. (2020) stated that mothers of autistic children have a wide role and involvement in the care of these children due to the poor performance of these children in self‐care (Mo et al., 2020). Also, Flippin and Crais (2011) stated that mothers of autistic children are primarily responsible for the care of these children in the family who have a high‐care load and active participation in child care, and there is a strong relationship between the burden of caring for autistic children and the participation of their mothers in care, which is in line with the findings of the present study (Flippin & Crais, 2011).

Coinciding with this study, Mello et al. (2019) also reported that mothers of children with autism are most involved in caring for these children, and that whatever the care burden for these children is more, their involvement in care is the wider (Mello et al., 2019).

Finally, it can be stated that according to the findings of this study, mothers of children with autism carrying a high burden in caring for their children and have moderate adaption to existing conditions but are still actively involved in caring of their autistic children. Therefore, it is necessary basic planning and extensive support to reduce caring burden and subsequently improve coping strategies in these mothers.

### 4.1 Limitations

One of the most important limitations of the present study was the non‐return of questionnaires. This is probably due to the busy mothers in the Corona crisis. On the other hand, the studied variables have been measured during 12 months involving with coronavirus. Accordingly, it is suggested to evaluate the care burden, coping strategies during corona in different societies and with larger sample size to achieve more accurately estimation about the care burden of autistic children' mothers in Corona crisis and subsequently, based on these findings, managers and policymakers can make more comprehensive measurements and planning for this crisis or similar crises.

## 5 CONCLUSIONS

The care burden for mothers of autistic children during COVID‐19 pandemic was reported high in this study. Also, coping strategies, involvement in care, mother's job, the number of children, age and functional level of autistic child affected the care burden imposed on these mothers. Accordingly, paediatric nurses and policymakers of health organizations can by providing a supportive environment; counselling and training these mothers promote the adaptation and participation them in caring of children with autism.

## AUTHOR CONTRIBUTIONS

FM, NS, SRB, SKH, EJ, SB, MR and MB were involved in the conception of the study and designed the study. They are responsible for data collection. Then FM, NS, SRB and SKH analysed data. FM, SZM and KHO drafted the primary manuscript, revised and approved the final manuscript.

## CONFLICT OF INTEREST

No conflict of interest is declared by the authors.

## ETHICAL APPROVAL

The institutional review board of the Hamadan University of Medical Science located in the west of Iran provided ethics approval (approval number: 1400.171) with research project number (140004082980), Also at the beginning of each interview, the researcher introduced herself and explained the goals of the study and assured that all information would remain confidential and that they could withdraw from the study at any time. The researchers provided the opportunity for participants to inform the researcher about their withdrawal from the study at any stage of the research and assured. Finally, written consent was obtained from study participants.

## Data Availability

The data sets used and/or analysed during the current study are available from the corresponding author on reasonable request.
